# Preterm Labor and Chorioamnionitis Are Associated with Neonatal T Cell Activation

**DOI:** 10.1371/journal.pone.0016698

**Published:** 2011-02-08

**Authors:** Angel A. Luciano, Haiyan Yu, Leila W. Jackson, Lisa A. Wolfe, Helene B. Bernstein

**Affiliations:** 1 Department of Pediatrics, Division of Neonatology, University of South Florida, College of Medicine, Tampa, Florida and USF Children's Research Institute, St. Petersburg, Florida, United States of America; 2 Department of Reproductive Biology, Case Western Reserve University School of Medicine, Department of Obstetrics and Gynecology, MacDonald Women's Hospital, University Hospitals Case Medical Center, Cleveland, Ohio, United States of America; 3 Department of Obstetrics and Gynecology, West China Second University Hospital, Sichuan University, Chengdu, China; 4 Department of Epidemiology and Biostatistics, Case Western Reserve University School of Medicine, Cleveland, Ohio, United States of America; The University of Queensland, Australia

## Abstract

**Background:**

Preterm parturition is characterized by innate immune activation and increased proinflammatory cytokine levels. This well established association leads us to hypothesize that preterm delivery is also associated with neonatal T lymphocyte activation and maturation.

**Methodology/Principal Findings:**

Cord blood samples were obtained following term, preterm, and deliveries complicated by clinical chorioamnionitis. Activation marker expression was quantitated by flow cytometric analysis. Infants born following preterm delivery demonstrated enhanced CD4^+^ T lymphocyte activation, as determined by CD25 (Term 9.72% vs. Preterm 17.67%, p = 0.0001), HLA-DR (Term 0.91% vs. Preterm 1.92%, p = 0.0012), and CD69 expression (Term 0.38% vs. Preterm 1.20%, p = 0.0003). Neonates delivered following clinical chorioamnionitis also demonstrated increased T cell activation. Preterm neonates had an increased frequency of CD45RO^+^ T cells.

**Conclusion/Significance:**

Preterm parturition is associated with neonatal CD4^+^ T cell activation, and an increased frequency of CD45RO^+^ T cells. These findings support the concept that activation of the fetal adaptive immune system in utero is closely associated with preterm labor.

## Introduction

Preterm birth impacts up to 12% of all deliveries, is the leading cause of neonatal morbidity and mortality, and accounts for approximately one half of long-term neurologic morbidity in children. While advances in medicine have improved the survival of premature infants, the rate of preterm birth in the United States has not decreased [1], [2]. This has led to an increased prevalence of surviving preterm children with handicaps. In spite of its importance, the etiology of preterm labor is not well understood. Intrauterine infection is strongly linked to birth prior to 30 weeks [3], [4]. However, diverse factors including inflammation, race, environmental stress, and genetics are also associated with preterm labor.

Several lines of evidence support the association of infection with preterm labor. Intrauterine infection or the systemic administration of lipopolysaccharide (LPS) to pregnant animals can result in preterm labor and delivery [5], [6], [7], [8]. Maternal, non-obstetrical infections such as pyelonephritis, pneumonia, and periodontal disease are also associated with preterm birth, implying that systemic inflammation can lead to preterm parturition. Studies demonstrating that antibiotics prolong gestation following preterm, premature rupture of membranes, with an increased latency period and improved neonatal outcome within antibiotic-treated women, support a link between infection, inflammation and preterm birth [9], [10].

There is a well-described association between fetal inflammation and preterm birth. The fetal inflammatory response syndrome (FIRS) was originally described by Gomez, et al in 1998, characterized by increased cord blood IL-6 levels, and associated with a high rate of severe neonatal morbidity including respiratory distress syndrome, neonatal sepsis, bronchopulmonary dysplasia, intraventricular hemorrhage, periventricular leukomalacia and necrotizing enterocolitis [11]. Acute inflammation is initially mediated by the innate immune system, producing vasodilation, complement activation, and the release of inflammatory mediators including cytokines and chemokines. Subsequently, as inflammation progresses, the adaptive immune system becomes involved via recruitment by soluble factors, innate immune cells, Toll-like receptor ligands, and antigen presenting cells [6], [12]. Although several investigators have assessed the role of innate immune activation in preterm parturition [11], [13], [14], much less is known about the role of T cell activation in preterm delivery.

T cell activation can be assessed by the presence of activation markers on the cell surface, furthermore T cell activation in the presence of cognate antigen leads to the development of memory CD45RO^+^ T cells [15]. To define the role of fetal lymphocyte activation in preterm birth we assessed neonatal CD4^+^ T lymphocyte activation and memory phenotype *ex viv*o utilizing fluorescently labeled antibody staining followed by flow cytometric analysis. This permits simultaneous identification of the cell type and the activation state in addition to other phenotypic markers; activation markers assessed include: **CD69**, **CD25, HLA-DR** and **CD45RO**. This methodology was previously used, whereby we found that spontaneous labor at term is not associated with neonatal T cell activation [16]. In this study we examined the association between preterm birth neonatal T lymphocyte activation, as well as levels of memory CD45RO^+^ T cells to determine whether there is a link between the fetal adaptive immune response and preterm birth.

## Results

### Preterm birth is associated with neonatal lymphocyte activation

**Figure 1:**
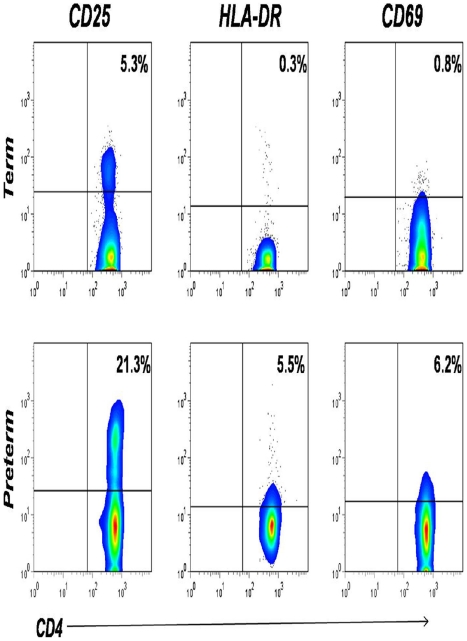
Cell surface activation marker expression on neonatal CD4^+^ T cells. Cord blood samples obtained simultaneously from an uncomplicated term delivery (control) and a delivery complicated by preterm labor were stained with fluorochrome labeled antibodies for cell surface activation markers (CD25, HLA-DR, and CD69, y axis of each dot plot) and subjected to three-color flow cytometric analysis. Cells were gated based on size and CD3 expression, quadrants were set based on isotype controls, and samples were compensated electronically for overlap in fluorescent emission. The percentage of cells in each quadrant is indicated. These data are representative of 34 term infants and 12 preterm infants.

**Figure 2:**
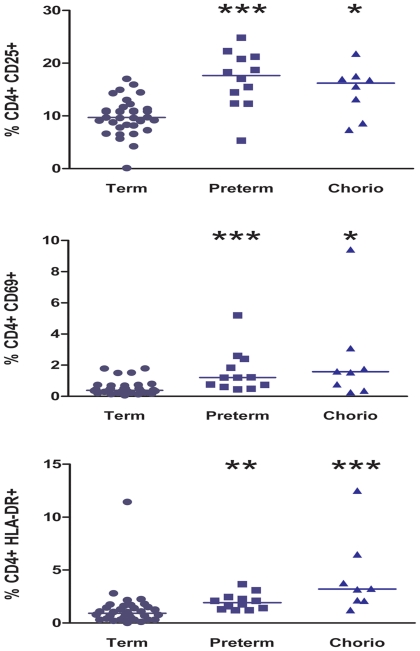
Preterm neonates have increased T cell activation. Cord blood samples from idiopathic preterm and uncomplicated term deliveries were stained with labeled antibodies for cell surface activation markers (CD25, CD69, and HLA-DR) and subjected to flow cytometric analysis. Median CD25 expression in term infants (n = 34) was 9.72% (Interquartile range (IQR): 8.18, 11.30), 17.67% (IQR: 13.40, 21.02) in preterm infants (n = 12), and 16.21 (IQR: 10.91, 17.26) in infants with chorioamnionitis (n = 8). Median CD69 expression was as follows: 0.38% (IQR: 0.29, 0.70) in term infants, 1.20% (IQR: 0.67, 2.12) in preterm infants, and 1.58% (IQR: 0.56, 2.42) in infants with chorioamnionitis, Median HLA-DR expression was as follows: 0.91% (IQR: 0.40, 1.50) in term infants, 1.92% (IQR: 1.36, 2.36) within preterm infants, and 3.21% (IQR: 2.11, 5.13) within infants with chorioamnionitis (* represents p-value <0.05, ** represents *P*-value <0.01, and *** represents p-value <0.001 for the comparison to term infants). Preterm infants and infants with chorioamnionitis had significantly different HLA-DR expression (*P* = 0.045), but not CD25 or CD69 expression. Activation marker data not available for 3 preterm infants' samples.

T lymphocytes were identified in cord blood samples based on cell size (forward scatter) and cellular complexity or granularity (side scatter), and CD3 expression. To evaluate the effect of preterm delivery on neonatal T cells, whole blood was stained *“ex vivo”* using monoclonal fluorochrome-labeled antibodies. This permits simultaneous identification of the cell type and the activation state in addition to other phenotypic markers. We hypothesized that preterm neonatal CD4^+^ T cells might demonstrate an activated phenotype expressing elevated levels of CD25, HLA-DR and CD69. Following deliveries complicated by preterm labor, there was an increased percentage of CD4^+^ T cells expressing activation markers, as measured by flow cytometry compared to uncomplicated term deliveries (Figure 1). Infants from pregnancies with a documented case of clinical chorioamnionitis prior to or at the time of delivery were excluded from this analysis. Preterm infants had a statistically significant increase in CD25 (P = 0.0001), CD69 (P = 0.0003) and HLA-DR (P = 0.0012) expression on CD4^+^ T cells compared to term neonates (Figure 2); significant differences remained when outliers were excluded from statistical analysis (data not shown).

### Clinical chorioamnionitis is also associated with neonatal T cell activation

After finding significant neonatal T cell activation following preterm delivery, we assessed T cell activation in infants delivered to mothers with a diagnosis of clinical chorioamnionitis, defined as the presence of maternal fever, tachycardia, and uterine tenderness and a decision by the treating physician to institute intrapartum parenteral antibiotics. This group included both term and preterm infants. We found that infants delivered in the setting of chorioamnionitis also demonstrated significant T cell activation, with increased CD25 (P = 0.0149), CD69 (P = 0.0104), and HLA-DR expression (P = 0.0004), compared to term infants without clinical chorioamnionitis (Figure 2); significant differences remained when outliers were excluded from statistical analysis (data not shown). When T cell activation within the group of preterm infants was compared to the group of infants born to mothers with chorioamnionitis, there was no difference in CD25 or CD69 expression. Infants delivered in the presence of chorioamnionitis (3.21%; IQR: 2.11, 5.13) had greater HLA-DR expression than did preterm neonates (1.92%, IQR: 1.36, 2.36; P = 0.04), when outliers were excluded from analysis this difference was no longer significant (P = 0.09, data not shown). Both of these groups displayed significantly increased T cell activation, compared to normal, term infants.

### Preterm birth is associated with an increased frequency of CD45RO^+^ T cells in the neonate

**Figure 3:**
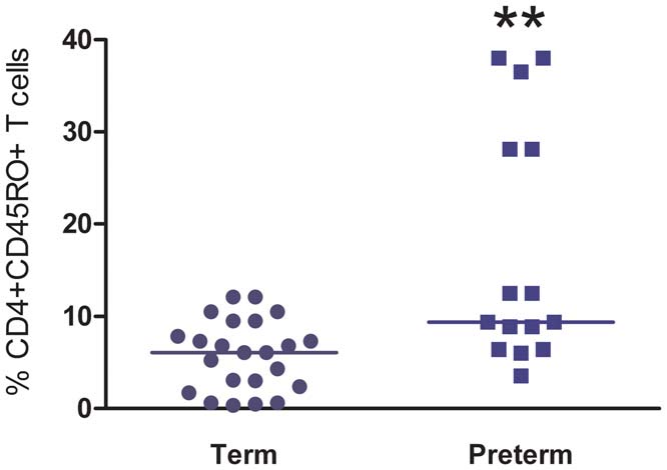
Preterm infants demonstrate enhanced memory T cell induction. Memory T cell frequency was compared between the term group (n = 23) and the preterm group (n = 15). Preterm neonates had an increased median percentage of memory CD4+ T cells (9.40%, IQR: 6.40, 28.10), CD45R0+/CD45RA-, compared to the median percentage of memory CD4+ T cells in term infants (6.10%, IQR: 2.37, 9.50) in term infants (*P* = 0.0032).

Antigen specific activation is involved in the formation of CD45RO^+^ T cells [15]. As neonates have limited antigen exposure, we expected to find low numbers of CD45RO^+^ T cells. Following our observation that CD4^+^ T cell activation occurs in neonates delivered preterm, we assessed memory (CD45RO^+^) and naïve (CD45RA^+^) T cell percentages in term and preterm neonates. We found that preterm neonates had an increased median percentage of memory CD4^+^ T cells (9.40%; IQR: 6.40, 28.10), as assessed by CD45RO expression and the absence of CD45RA expression (Figure 3), compared to the median percentage of memory CD4^+^ T cells within term infants (6.10% IQR: 2.37, 9.50; P = 0.0032).

## Discussion

Preterm parturition is associated with fetal and neonatal inflammation. Several researchers have described increased neonatal serum cytokine levels following preterm delivery; [11], [12], [17], [18], [19], [20], [21] however, it is not understood whether the inflammation originates from the mother, fetus, or both. There is also evidence that innate immune cells are activated at the time of preterm birth; whereby investigators have described a fetal inflammatory response syndrome associated with preterm parturition [11], [13], [14]. Although acute inflammation is typically mediated by the innate immune system, established inflammation potentially involves both the innate and adaptive immune system. Most studies to date assessing neonatal immune activation have focused on the innate immune system. To better understand if preterm parturition is associated with adaptive immune activation, we assessed neonatal T cell activation following idiopathic preterm birth and uncomplicated term birth.

Following deliveries complicated by preterm labor, there was increased expression of the activation markers CD25, HLA-DR, and CD69 on CD4^+^ T cells within cord blood as measured by flow cytometry. For all activation markers assessed there was a statistically significant increase, when preterm neonates were compared with term neonates. Additionally, gestational age at the time of spontaneous birth was inversely correlated with T cell activation in the absence of clinical chorioamnionitis (data not shown). These findings suggest that early preterm delivery is associated with higher levels of immune activation which is in agreement with other studies that have shown that early preterm delivery is more likely to be secondary to an infectious etiology [3], [4] To confi.rm that our observations are related to inflammation and not secondary to gestational age, we need to assess T cell activation in gestational age matched infants delivered for indications which do not involve inflammation, studies are ongoing to address this issue.

Our findings support the concept that the fetal adaptive immune response and preterm labor are linked. As lymphocytes produce cytokines upon activation, activated T cells may be responsible, in part, for the increased fetal cytokine levels found in association with preterm birth. T cell activation in the setting of antigen or cytokine exposure can also lead to the development of memory T cells, characterized by the presence of CD45RO expression and the absence of CD45RA expression. As memory cells produce significantly more cytokines than do naïve T cells, increased levels of memory T cells could further contribute to the increased and rapid neonatal serum cytokine levels seen in the setting of preterm delivery. Investigators have observed an increased frequency of CD45RO^+^ T cells following deliveries complicated by intrapartum infection [22], [23]. We determined the frequency of CD4^+^ memory T cells in neonates born preterm, in the absence of clinical chorioamnionitis, and found that they had an increased percentage of memory T cells compared to infants born at term without complications. This correlated with a previously published study that found preterm neonates had fewer naïve T cells [24].

The present study extends the work of other investigators, who have described innate immune activation following preterm birth [13], [14]. Our observation that preterm delivery is associated with T cell activation, suggests that the inflammation associated with preterm parturition is well established, as acute inflammation is more typically restricted to the innate immune system. Expansion of inflammation to involve the adaptive immune system, might occur via soluble factors, professional antigen presenting cells, ligation of Toll-like receptors or a combination of factors [25]. It is not clear if this phenomenon initiates a cascade of events that predisposes to preterm parturition or if this immune activation is the result of preterm labor. Genetic predisposition and environment interactions also potentially modulate immune activation, these possibilities are currently under investigation.

Infants born to mothers with clinical chorioamnionitis defined as a temperature more than 100.4°F, tachycardia, and uterine tenderness requiring antepartum and/or intrapartum antibiotics, also demonstrated T cell activation. Interestingly, T cell activation within preterm neonates was similar to infants delivered following chorioamnionitis. Both of these conditions were associated with significantly more T cell activation, compared to neonates following normal, term birth. These findings demonstrate an association between fetal lymphocyte activation, chorioamnionitis, and preterm labor; and support the concept that preterm parturition and chorioamnionitis are associated with significant immune activation and inflammation, impacting both the innate and adaptive immune systems. Further studies are needed to better understand whether fetal/neonatal immune activation mediates preterm birth, or is a result of preterm labor.

The role of the maternal immune system in preterm parturition also warrants further study. Although cytokines clearly mediate immune activation, it is not clear whether they originate from the maternal or fetal compartment or how easily cytokines cross materno-fetal boundaries. Moreover, little is known about maternal immune activation in the setting of preterm parturition. Identifying the source of cytokines associated with preterm birth, and their influence on both maternal and neonatal immune function, will increase our understanding of the immunopathogenetic processes associated with preterm birth.

## Materials and Methods

### Subjects

Cord blood samples were obtained from patients delivering at University Hospitals of Cleveland/MacDonald's Women Hospital and The Johns Hopkins Hospital after obtaining written informed consent. The study was approved by the Institutional Review Boards of both institutions. Samples were obtained from term infants born without maternal complications (gestational age equal to or greater than 37 weeks, n = 34), preterm infants without clinical chorioamnionitis delivered following spontaneous, idiopathic preterm labor (gestational age less than 35 weeks, n = 15), and preterm and term infants delivered from mothers with documented, clinical chorioamnionitis, defined as maternal fever (Temperature above 100.4°F), tachycardia, and uterine tenderness requiring antibiotic treatment (n = 8). Exclusion criteria included: pre-eclampsia, premature rupture of membranes, congenital anomalies, significant maternal illness, maternal or fetal indications for delivery, and prostaglandin induction of labor. Demographic and clinical details from patients delivering at University Hospitals were obtained from the medical record (Table 1); only gestational age, mode of delivery and presence or absence of clinical chorioamnionitis was documented from anonymous specimens from patients delivering at Johns Hopkins Hospital, secondary to the IRB protocol in place at the time of sample collection. Possible causes for preterm labor and delivery were examined.

**Table 1:** Maternal and fetal characteristics.

Demographics	Term (n = 17)	Preterm (n = 12)	p value
Mean maternal age (SD)	*27.7 (+/-7.2)*	*26.7 (+/-8.0)*	*0.73*
Mean gestational age in weeks (SD)	*38.7 (+/-1.3)*	*29.1 (+/-2.7)*	*>0.001*
Mean birth weight in grams (SD)	*3107 (+/- 632)*	*1377 (+/- 463)*	*>0.001*
Median APGAR 1 min (IQR)	*9 (8,9)*	*5.5 (2,7)*	*0.001*
Median APGAR 5 min (IQR)	*9 (9,9)*	*7 (6,8)*	*>0.001*
Prenatal steroids (%)	*N/A*	*12(100%)*	
Prenatal antibiotics (%)	*1(6%)*	*10(83%)*	
Prenatal magnesium sulfate (%)	*0*	*8(67%)*	

### Cell preparation and flow cytometry

Cord blood from term and preterm pregnancies was collected according to the National Cord Blood Program collection protocol. Activation was assessed in whole blood within 4 hours of delivery; this rapid analysis permits accurate assessment of the in vivo activation state. Minimal sample processing and rapid *ex vivo* analysis yields results reflecting the *in vivo* activation state.

For each stain, 100 µL of blood was placed in a fluorescence-activated cell sorter tube, washed once, and incubated for 15 minutes with 20 µL of human AB serum for blocking. Conjugated antibodies were then added to each tube and incubated for 15 minutes at room temperature as described [16]. Erythrocytes were lysed and cells fixed with FACSlyse (BD Biosciences). Four-color blood flow cytometric analysis was performed on a FACScalibur (BD Biosciences); lymphocyte gating was based on size, granularity, and CD3 expression. At least 5000 gated events were obtained for analysis; FlowJo (version 7 Tree Star, Ashland,OR), was used to organize and analyze the data.

### Statistical analysis

The Shapiro-Wilk normality test was used to assess the distribution of data using an alpha value of 0.05. Based on normality, group means or medians were compared using either a Student's t-test or Wilcoxon Rank Sum test; a p-value of 0.05 was considered nominally significant. Data was analyzed using Stata11.0 (StataCorp LP, College Station, TX) and Prism 5.0 Graphpad software (La Jolla, CA).
